# AstigMATIC: an automatic tool for standard astigmatism vector analysis

**DOI:** 10.1186/s12886-018-0920-1

**Published:** 2018-09-21

**Authors:** Mathieu Gauvin, Avi Wallerstein

**Affiliations:** 1 LASIK MD, Montreal, QC Canada; 2 Department of Ophthalmology, Faculty of Medicine, McGill University, Montreal, QC Canada

**Keywords:** Astigmatism vector analysis, Target-induced astigmatism vector, Surgically-induced astigmatism vector, Difference vector, Correction index, Alpins Method

## Abstract

**Background:**

Standardization for reporting medical outcomes facilitates clinical study comparisons and has a fundamental role on research reproducibility. In this context, we present AstigMATIC, a free standalone application for automated standardized astigmatism vector analyses in corneal and intraocular refractive surgeries. AstigMATIC uses a simple graphical user interface (GUI) and allows the simultaneous display and analysis of astigmatism magnitude and axis.

**Results:**

The software produces the four following standard graphs according to the standards of the Alpins Method; 1-Target-Induced Astigmatism Vector, 2-Surgically-Induced Astigmatism Vector, 3-Difference Vector and 4-Correction Index. Vector means with X and Y standard deviations are automatically calculated and displayed on the corresponding single-angle vector plots (0 to 180°). Data points are entered into a simplified GUI with no need for command line input. The standard graphs can be easily exported as high-resolution TIFF images for figures to use in production and presentations.

**Conclusions:**

AstigMATIC software enables the user to easily and efficiently analyze vectorial astigmatism outcomes using the standardized Alpins Method for post-surgical astigmatism. AstigMATIC and the demonstration datasets are available to download from http://www.lasikmd.com/media/astigmatic.

## Background

### Introduction

Standardization for reporting medical outcomes facilitates clinical study comparisons and has a fundamental role on reproducibility [1, 2]. In refractive surgery (RS), the first set of standards for outcomes reporting was originally proposed by Waring in 1992 [3] and later implemented as a set of six standard figures summarizing the accuracy, efficacy, safety, and stability of a surgical procedure [4–10]. It is now required to include these graphs for RS manuscripts submitted to the Journal of Refractive Surgery (JRS) [6], the Journal of Cataract and Refractive Surgery (JCRS) [7] and Cornea [8]. Other journals, such as Ophthalmology, also recommend the use of these standard graphs in their author guidelines [10]. Due to these specifications, the reporting of outcomes of particular surgical techniques, studies, case reports or case series are standardized, and results are easily comparable between and within RS studies [1].

The set of six figures was recently expanded to nine graphs in order to include additional information regarding astigmatism outcomes [1], and a similar set of standards was recently added to cover lens-based refractive surgery [2]. While these newer figures cover the main outcome measures for refractive surgery, in studies where astigmatism correction is a contributing feature, supplementary vectorial astigmatism analyses should be conducted [1, 2]. For astigmatism vector analyses, JRS [1], and JCRS [11] recommend using the Alpins Method [10, 12–15] reported as single-angle polar plots [1, 11]. The Alpins vector analysis method consists of a set of four graphs, and each answers specific questions related to astigmatism correction [1]. These four graphs allow a more detailed understanding of astigmatism outcomes pre- to post-surgery, and can better identify precision in surgical results [10, 12–15]. The Alpins Method lends itself to any ocular procedure where astigmatism outcomes need to be thoroughly assessed [10], such as PRK, LASIK, LASEK, SMILE, incisional keratotomy, collagen cross-linking, intracorneal ring segments, as well as lens-based procedures such as Phakic IOL, cataract surgery and refractive lens exchange with multifocal IOL and/or Toric IOL, as well as the various modified surgical techniques or procedures to treat various RS complications that exist today [16–25].

The six and nine standard graph formats described above can be created by downloading free macro-enabled Microsoft Excel spreadsheets [1, 2], as well as with paid web-based and desktop software specially designed for refractive surgery outcomes analysis. In contrast, an automated specialized freeware for the production of the four standard vector analysis graphs remains unavailable, thus limiting their widespread use. In this context, we present AstigMATIC, a free standalone executable application that produces the four standardized astigmatism vector graphs as per the latest standards of refractive surgery journals. AstigMATIC should help clinicians and researchers rapidly understand clinical outcomes using the Alpins Method and will provide them with vector graphs that respect current journals standards for research publications and presentations.

### Reporting and visualizing vectorial astigmatism outcomes

A growing number of journals and authors agree that reports including vectorial astigmatism should adhere to the vectorial astigmatism nomenclature first described by Alpins [10, 12–15]. The Alpins Method has been used for the last 25 years and has become a widely-accepted approach in the field with several hundred peer-reviewed publications utilizing it [10, 12–15]. The Alpins Method graphs include the following:

1. Target-induced astigmatism (TIA) vector: this graph shows the range of astigmatism (magnitude and axis) that the surgery intended to induce.
2. Surgically-induced astigmatism (SIA) vector: this graph shows the range of achieved astigmatism cylinder and axis treatment and is used to compare the achieved (SIA) astigmatism treatment to the intended (TIA) treatment.
3. Difference vector (DV): this graph shows remaining astigmatism and provides a summary of the astigmatic error considering both magnitude and axis. The DV is often used as an absolute measure of success and is preferably null.
4. Correction index (CI): this graph shows the under/overcorrection of the astigmatism treatment. The CI can also be used as a measure of success and is calculated as SIA divided by TIA. With an optimal surgical outcome it is equal to 1, and is greater and smaller than 1 if an overcorrection and undercorrection occurs, respectively.

Based on these well-established metrics [10, 12–15], AstigMATIC provides the automated production of these four recommended single-angle polar graphs. Advantages of using single-angle polar plots vs double-angle plots is that they are easily transferable to a clinical situation with corneal topography, treatment parameters, or the eye itself. Single-angle plots are also efficient and require half the space of double-angle plots [1]. Journals will usually accept quality studies that employ either single-angle or double-angle representation, but given the above advantages, JRS and JCRS recommend the use of single-angle graphs. It is important to note that data points near 0° and 180° are not visually grouped and are on opposite sides on a single-angle polar plot. Ophthalmologists are familiar with single-angle polar plots and recognize that points near 0° are similar to points near 180° [1]. To help visualize and group points within the same area, we added, as previously proposed elsewhere [1], a shaded region to highlight the areas of common orientation. The blue regions, spreading from 0 to 30 degrees and from 150 to 180 degrees, highlight against-the-rule astigmatism, the red region, spreading from 60 to 120 degrees, highlights within-the-rule astigmatism, while unshaded white regions, spreading from 30 to 60 degrees and from 120 to 150 degrees, highlight oblique astigmatism.

Of note, there are a limited number of papers that use the American National Standards Institute (ANSI) nomenclature [26], but the use of this nomenclature is incorrect and its use should be avoided [10]. AstigMATIC uses the Alpins’ nomenclature as it remains the most widely used terminology and the only one that should be used in the field [10, 12–15].

## Implementation

### Software implementation and system requirements

AstigMATIC is programmed in MATLAB R2018a (Mathworks Inc., Natick, MA, USA) and compiled using the MATLAB runtime compiler (Mathworks Inc.). Thus, AstigMATIC is available as an executable that can be run independently of MATLAB installation and without a MATLAB license, providing that the associated MATLAB runtime compiler (MRC) is correctly installed on the computer before running the AstigMATIC executable. AstigMATIC has been tested on Windows 7 Enterprise and Professional and Windows 10 Home and Professional, all with a 64-bit-operating system, and with a 1920 × 1080 screen resolution. AstigMATIC and the demonstration datasets are available to download from http://www.lasikmd.com/media/astigmatic.

### Input data format

To automatically generate the figures, AstigMATIC reads data files in the Microsoft Excel format (e.g. Datafile.xlsx). This file format was selected due to its widespread use and simplicity. There are five columns. The first column is the eye reported as “OD” or “OS” (i.e., for the right and left eye, respectively), the second and third columns are the preoperative cylinder magnitude and axis, respectively, and the fourth and fifth columns are the post-operative cylinder magnitude and axis, respectively (Table 1). The data must be provided in the point decimal format (e.g. -1.50, 0.75, etc.). Both positive cylinder (+ve) and negative cylinder (-ve) nomenclature can be used. AstigMATIC does not convert the subjective refractive astigmatism, generally measured at a vertex distance of 12mm, to the corneal plane at 0 mm, and will not auto-discard eyes with 0.00 D of astigmatism. Data conversion and exclusion is at the user’s discretion prior to data importation. Rows with missing data are automatically discarded from the analyses.

**Table 1:** Data file format example

	Eye	Preop Magnitude	Preop Axis	Postop Magnitude	Postop Axis
Data point 1	OD	0.25	007	0	0
Data point 2	OS	0.75	88	−0.25	^a^
Data point 3	OD	−2.00^b^	90	0	0
…	…	…	…	…	…
Data point N	OS	−1.00^b^	180	0	0

Example of the Excel data file format that is read by AstigMATIC to generate the four standard astigmatism vector graphs. Note that the first column and row should not be included in the actual data file. Please use the point decimal notation and not comma. Three data file samples are provided at http://www.lasikmd.com/media/astigmatic/

^a^ This row will automatically be discarded given the missing value

^b^ This row will automatically be converted from -ve cylinder to +ve cylinder format. For example, − 2.00 × 90 will be converted to + 2.00 × 180 and − 1.00 × 180 to + 1.00 × 90

### Program workflow

**Fig. 1:**
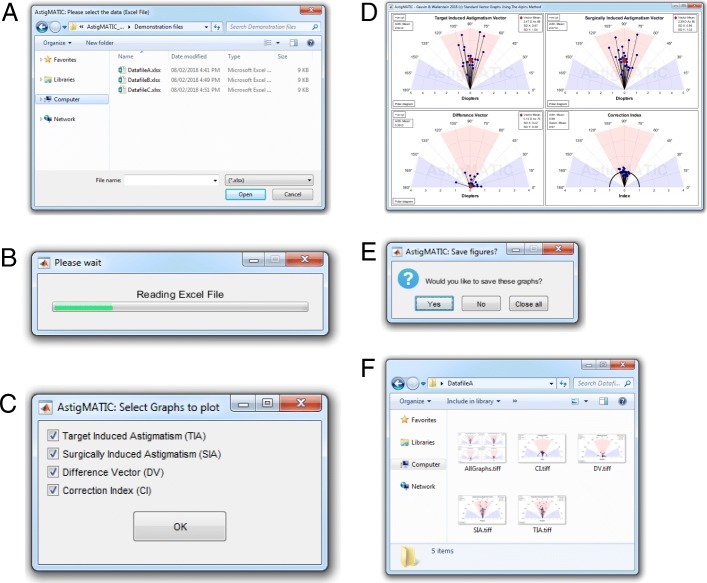
Flow chart of the AstigMATIC workflow. **a-b** Upon starting the application, raw astigmatism data must first be imported. **c** The user is invited to select which graphs to include in the analysis. **d** The selected graphs are then generated. **e** The user is invited to visualize, save or close the figures. **f** By selecting “save”, the figures are automatically saved as TIFF images in a folder with the same name and location as the original Excel data file

The flow chart of the AstigMATIC workflow is shown in Fig. 1. The application was designed to be user-friendly and to be accessible to users with no knowledge of MATLAB coding. To this end, the application is entirely controlled via a few simple steps that are triggered as the user progresses through the program workflow (Fig. 1). Upon starting the application, raw astigmatism data must first be imported (Fig. 1a-b). Once the data file is selected (see example in Table 1), the user is invited to select which graphs to include in the analysis (Fig. 1c). By default, the four graphs are selected (Fig. 1c). The selected graphs are then generated (Fig. 1d) and the user invited to visualize, save or close the figures (Fig. 1e). By selecting “save”, the figures are automatically saved as TIFF images in a folder with the same name and location as the original Excel data file (Fig. 1f).

### Usage

AstigMATIC can be used to automatically produce the standard graphs for reporting outcomes for astigmatism correction, based on the Alpins Method, that are recommended by refractive surgery and ophthalmology journals [10, 12–15]. The tool was developed for teaching and academic research purposes in the context of laser refractive surgery, but its use can be extended to analyze any corneal and intraocular refractive surgery outcomes [16–25]. AstigMATIC can be used providing that the user(s) cite the current manuscript when including results generated from AstigMATIC in any publication, presentation, or other public communication, whether peer-reviewed or non-peer-reviewed.

### Calculation of astigmatism vectors

The four stigmatism vectors are calculated according to the Alpins Method which was previously, and extensively, described elsewhere [10, 12–15]. Briefly, prior to vectorial analyses, negative cylinder (i.e., −ve) values are converted to positive cylinder (i.e., +ve) by flipping the axis by 90 degrees and by taking the absolute value of the magnitude. For example, − 1.00 × 180 is converted to + 1.00 × 90. AstigMATIC also flips the astigmatism axis of left eyes (OS) around the vertical axis so that errors induced by cyclotorsion or unsymmetrical healing do not have a tendency to cancel out when averaging data from both eyes. The TIA and SIA vectors are then calculated by converting the pre-operative and post-operative astigmatism values from polar coordinates to rectangular coordinates using basic trigonometry, as extensively described elsewhere [10–14]. When the target is emmetropia, the TIA magnitude is essentially equal to the pre-operative magnitude. The SIA magnitude is obtained by subtracting the post-operative values from the pre-operative astigmatism values, in rectangular coordinates, in both X and Y directions and by combining the obtained X and Y SIA (*SIA*_*X*_, *SIA*_*Y*_) value using the square root of their summed squared values ($SIA=\sqrt{{SIA_X}^2+{SIA_Y}^2}$). The TIA and SIA calculations are followed by the calculation of the DV (i.e., TIA - SIA; vectorial difference) and CI (i.e., SIA / TIA) vectors. These vectors are then used to generate the four standard graphs, where the vector means are plotted as a red diamond, and where the arithmetic means and standard deviations for the X and Y directions are displayed in the call-out boxes, as previously proposed [1]. The interested reader can consult previous literature for more detailed formulas and calculations [10, 12–15].

## Results and discussion

**Fig. 2:**
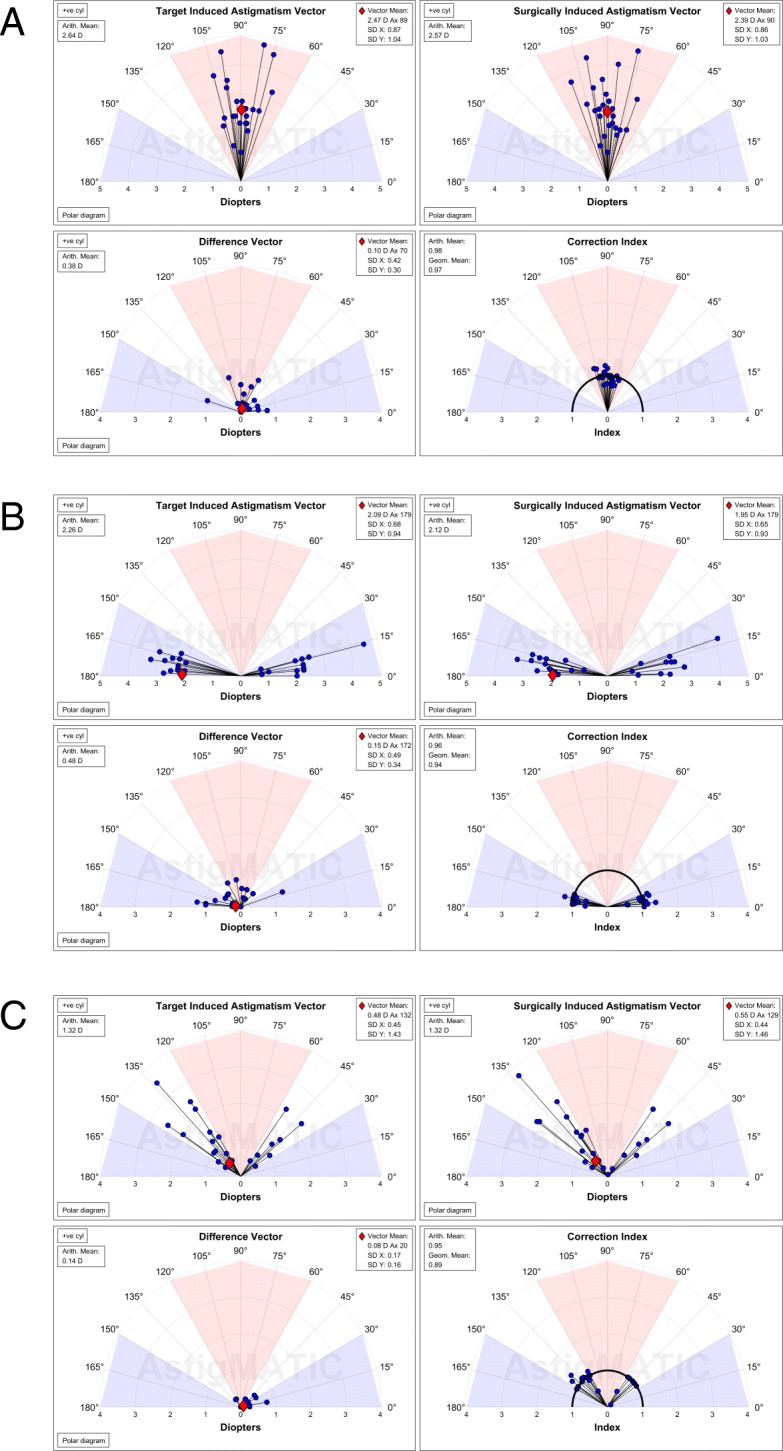
Examples of the four vector graphs that are generated by AstigMATIC from the pre- and post-operative cylinder magnitude and axis in within-the-rule (WTR) astigmatism eyes (**a**), against-the-rule (ATR) astigmatism eyes (**b**), and oblique astigmatism eyes (**c**). The TIA and SIA data points of WTR, ATR and oblique cases are located in the red, blue and white regions of the graphs, respectively. The red diamonds indicate the vector mean position. The vector mean values are displayed in the call-out boxes along with the arithmetic means or geometrical mean. Each individual vectorial point is plotted as a black line ending by a blue circle marker

A total of 3 simulated, realistic, datasets were produced by an experienced ophthalmic surgeon (A.W.) to test AstigMATIC. The first dataset included with-the-rule astigmatism cases, the second dataset comprised against-the-rule (ATR) astigmatism cases and the last dataset contained oblique astigmatism cases. Each dataset included 25 eyes. In each case, AstigMATIC was used to read the dataset (Excel files) and to automatically generate the four vector graphs (Fig. 2) from the pre- and post-operative cylinder magnitude and axis. As shown in Fig. 2a, b and c, the TIA and SIA data points of WTR, ATR and oblique cases are located in the red, blue and white regions of the graphs, respectively. These shaded areas help in rapidly identifying the type of astigmatism that was treated and could be used to highlight differences between these types of astigmatism. The red diamonds (Fig. 2a) indicate the vector mean position and are not reported in the CI graphs, as per standards [1]. The vector mean values are displayed in the call-out boxes along with the arithmetic means or geometrical mean. Each individual vectorial point is plotted as a black line ending by a blue circle marker, as per standards [1]. Values in diopters (top panels and bottom-left panel) are indicated at the bottom of each graphs, while the CI graphs shows the index values (bottom-right panel). Axis values are indicated in degrees around each graph, from 0 (right side of the graphs) to 180 (left-side of the graphs).

### Limitations

The main advantage of AstigMATIC is that it provides automated reporting of standardized astigmatism vector outcomes through the utilization of a user-friendly GUI, and that no special skills or MATLAB knowledge is needed to use it. With this advantage comes its main limitation: the very nature of having an executable application, such as AstigMATIC, instead of individual MATLAB scripts is that it has a lack of flexibility. Changing the color of graphs, and/or fonts is not supported. Unlike individual MATLAB scripts (m-files), AstigMATIC cannot be easily manipulated, and those that want to create more customized figures will likely prefer to create their own custom scripts to analyze astigmatism data. We elected to fix the format and color of the AstigMATIC graphs so that they would be in accordance with current journal standards [1, 11]. The latter should further facilitate comparisons between studies as previously suggested elsewhere [1]. AstigMATIC is currently limited to astigmatism vector analyses and will not analyze other refractive errors, such as sphere or spherical equivalent. It cannot be used to specifically calculate corneal meridian changes induced by cataract or corneal incisions.

### Improvements and future work

AstigMATIC is currently limited to the creation of vector plots and does not currently have a function to generate vector analysis tables. Future versions might eventually include the automated generation of tables. Further plots (including double-angle plots) could also be added if needed. Please note that AstigMATIC do not currently compute any statistical test. The interested user can use the calculated averages and standard deviations to derive his own hypothesis testing.

### Significance of the AstigMATIC software

Astigmatism analyses are multi-dimensional and subtle nuances cannot be fully captured in a single graphical display [10, 12–15]. The Alpins Method provides a simple approach whereby four graphs are used to answer distinct questions, enabling the cause of an inaccurate astigmatic correction to be understood and the effectiveness of an astigmatic treatment to be evaluated [10, 12–15]. Free specialized software for automated standardized astigmatism vector analyses in corneal and intraocular refractive surgeries remains unavailable, thus preventing some authors from including these advanced analyses in their studies. We developed AstigMATIC, an automated easy-to-use free program, designed to analyze astigmatism outcomes of various refractive surgeries using single-angle polar plots, as per the latest standards prescribed by JRS [1], and JCRS [11]. For additional astigmatism analyses, more advanced paid-software are available, such as ASSORT (www.assort.com), which also include surgical planning tools.

## Conclusions

With AstigMATIC software, we provide a freely downloadable tool for detailed reporting of astigmatism treatment outcomes that can be used by clinicians and researchers to easily display standardized vectorial astigmatism outcomes for publication, presentation or clinical knowledge. AstigMATIC and the demonstration datasets are available to download from http://www.lasikmd.com/media/astigmatic.

## Abbreviations

ATR: Against-the-rule
CI: Correction Index
DV: Difference Vector
IOL: Intra-Ocular Lens
JCRS: Journal of Cataract and Refractive Surgery
JRS: Journal of Refractive Surgery
LASEK: Laser Epithelial Keratomileusis
LASIK: Laser-Assisted in Situ Keratomileusis
LRS: Laser Refractive Surgery
MRC: Matlab Runtime Compiler
PRK: Photorefractive Keratectomy
SIA: Surgically-Induced Astigmatism
SMILE: Small Incision Lenticule Extraction
TIA: Target-Induced Astigmatism
WTR: Within-the-rule
